# Frequent Occurrence of Mitochondrial DNA Mutations in Barrett’s Metaplasia without the Presence of Dysplasia

**DOI:** 10.1371/journal.pone.0037571

**Published:** 2012-05-22

**Authors:** Soong Lee, Moon-Jong Han, Ki-Sang Lee, Seung-Chul Back, David Hwang, Hwan-Young Kim, Jong-Hee Shin, Soon-Pal Suh, Dong-Wook Ryang, Hye-Ran Kim, Myung-Geun Shin

**Affiliations:** 1 Departments of Internal Medicine, College of Medicine, Seonam University, Namwon, Korea; 2 Departments of Laboratory Medicine, Chonnam National University Medical School and Chonnam National University Hwasun Hospital, Hwasun, Korea; 3 Brain Korea 21 Project, Center for Biomedical Human Resources, Chonnam National University, Gwangju, South Korea; 4 Environmental Health Center for Childhood Leukemia and Cancer, Chonnam National University Medical School and Chonnam National University Hwasun Hospital, Hwasun, Korea; Vanderbilt University Medical Center, United States of America

## Abstract

**Background:**

Barrett's esophagus (BE) is one of the most common premalignant lesions and can progress to esophageal adenocarcinoma (EA). The numerous molecular events may play a role in the neoplastic transformation of Barrett’s mucosa such as the change of DNA ploidy, p53 mutation and alteration of adhesion molecules. However, the molecular mechanism of the progression of BE to EA remains unclear and most studies of mitochondrial DNA (mtDNA) mutations in BE have performed on BE with the presence of dysplasia.

**Methods/Findings:**

Thus, the current study is to investigate new molecular events (Barrett’s esophageal tissue-specific-mtDNA alterations/instabilities) in mitochondrial genome and causative factors for their alterations using the corresponding adjacent normal mucosal tissue (NT) and tissue (BT) from 34 patients having Barrett’s metaplasia without the presence of dysplasia. Eighteen patients (53%) exhibited mtDNA mutations which were not found in adjacent NT. mtDNA copy number was about 3 times higher in BT than in adjacent NT. The activity of the mitochondrial respiratory chain enzyme complexes in tissues from Barrett’s metaplasia without the presence of dysplasia was impaired. Reactive oxygen species (ROS) level in BT was significantly higher than those in corresponding samples.

**Conclusion/Significance:**

High ROS level in BT may contribute to the development of mtDNA mutations, which may play a crucial role in disease progression and tumorigenesis in BE.

## Introduction

Patients with Barrett’s esophagus (BE) have a 30 to 125-fold higher risk of developing esophageal adenocarcinoma (EA) than those without this lesion. The presence of BE confers a 0.5∼1% per year risk of the development of EA [1]–[5]. The prevalence of esophageal cancer is increasing in the world and has a poor prognosis mainly because of presentation at late stage. Progression to esophageal cells to cancer follows the metaplasia-dysplasia-adenocarcinoma sequence. Barrett’s metaplasia of esophagus is defined as replacement of the normal squamous epithelium of distal esophagus by metaplastic glandular epithelium containing intestinal-type goblet cells. The numerous molecular events may play a role in the neoplastic transformation of Barrett’s mucosa such as the change of DNA ploidy, p53 mutation and alteration of adhesion molecules. However, the molecular mechanism of the progression of BE to EA remains unclear.

The mitochondrion is an important micro-organelle that produces the energy for cell development, differentiation and growth by inducing apoptosis [6]. mtDNA repair system is inefficient and there is a higher exposure to reactive oxygen species (ROS) produced in the process of adenosine triphosphate (ATP) synthesis. ROS are commonly released in chronic inflammatory tissue. mtDNA is one of the target of ROS and free radicals [7]. With inflammation and excess stress ROS can overwhelm the antioxidant system, which result in damage to mtDNA [6], [8]. Accumulation in mutations in mtDNA, leading to an impairment of mitochondrial function, has been implicated in the etiology of aging, several degenerative pathologies, and tumorigenesis based on the presence of novel hetero-and homoplasmies [9]. Somatic mtDNA mutations play a role in oncogenic transformation and they also can contribute to tumor progression by enhancing the metastatic potential of tumor cells [10]. Thus, the current study is to investigate new molecular events (Barrett’s metaplasia tissue-specific-mtDNA alterations/instabilities) in mitochondrial genome and causative factors for their alterations using the corresponding adjacent normal mucosal tissues and metaplasia tissue from Barrett’s metaplasia without the presence of dysplasia.

## Results

### mtDNA Sequence Alterations

**Figure 1:**
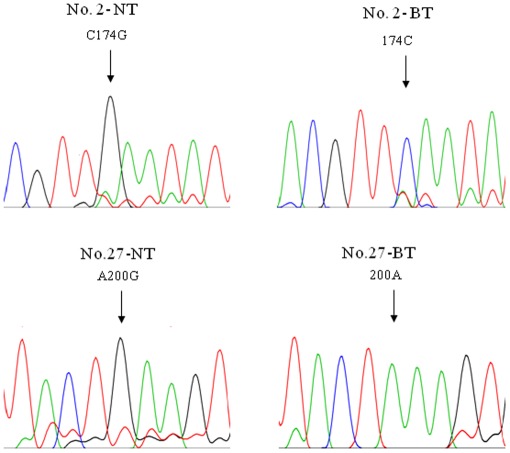
mtDNA substitution mutations only found in Barrett’s metaplasia tissue. Sequencing chromatograms showed Barrett’s metaplasia tissue (BT)-specific mtDNA mutations which were only observed in BT. Heteroplasmic mtDNA mutations were observed in *HV2* from patient’s No. 2 and 27. These heteroplasmic mutations were not found in the corresponding adjacent normal mucosal tissue (NT).

**Figure 2:**
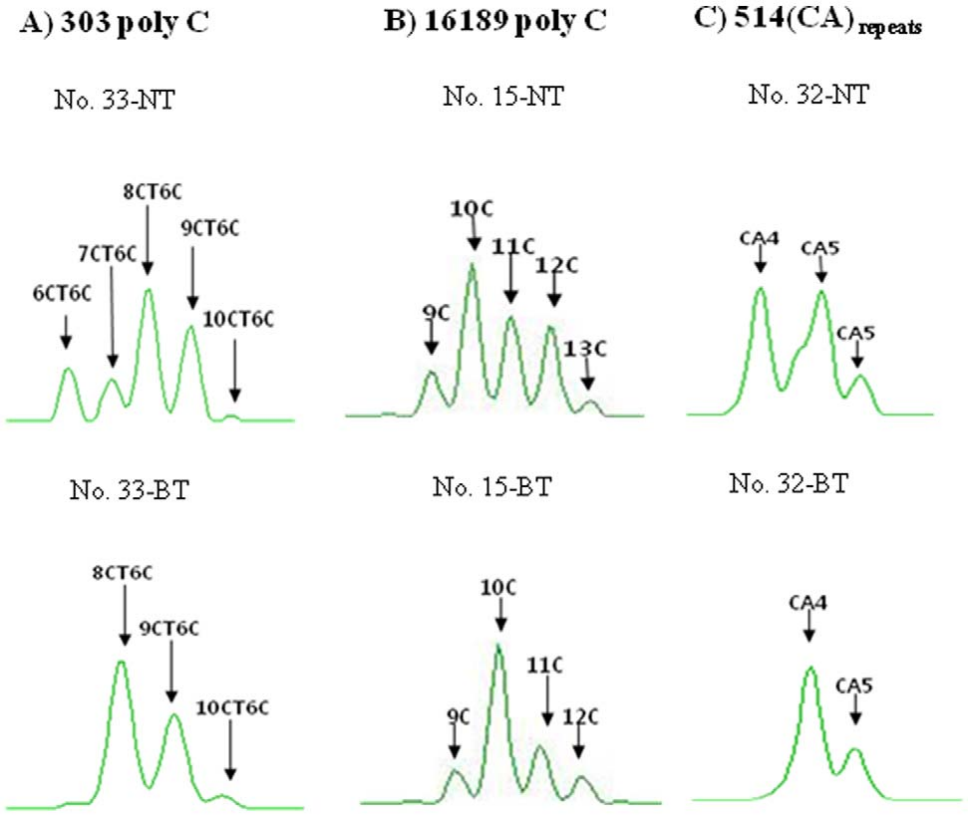
mtDNA length heteroplasmic mutations in Barrett’s metaplasia tissue. A gene scan analysis of the poly C stretch region at nucleotide position (np) 303 - 315 (A), np 16184 - 16193 (B) and (CA) repeat starting at np 514 (C) from Barrett’s metaplasia tissues (BT) compared to those from adjacent normal mucosal tissue (NT) demonstrated typical length heteroplasmic mutations in the poly C stretch regions from patient’s No. 33, 15 and 32 respectively.

In a set of analyses of the mtDNA control region obtained from adjacent NT and BT, characteristic heteroplasmic substitution mutations in the mtDNA control region were observed in two patients (6%), respectively (Table 1, Fig. 1). mtDNA length heteroplasmic mutation at poly C stretch of 303 poly C, 16189 poly C and 514 (CA) repeat were observed in 18 patients (53%) (Table 1, Fig. 2). Overall, BT-specific mtDNA mutations were observed in 53% (18/34) of all patients.

**Table 1:** mtDNA substitution and length heteroplasmic mutations in Barrett’s metaplasia tissue (BT) and adjacent normal mucosal tissue (NT).

Mutationstatus	mtDNAgenesorregions	Samples from patients with Barrett’s esophagus		PatientID	No.	%
		NT	BT	□	□	□
**Substitution**	Control/HV2	C174G/T		2	1	
	Control/HV2,HV1	A200G, T16136C, T16172C, A16183C		27	1	
	**Subtotal**				**2**	**5.9**
**Length hetero- plasmic** **mutation**	303 Poly C	8CT6C+9CT6C+10CT6C	8CT6C+9CT6C+10CT6C+11CT6C	1	1	
		8CT6C+9CT6C+10CT6C	7CT6C+8CT6C+9CT6C+10CT6C+11CT6C+12CT6C	2	1	
		7CT6C+8CT6C+9CT6C	7CT6C+8CT6C	5	1	
		8CT6C+9CT6C+10CT6C+11CT6C+12CT6C	8CT6C+9CT6C+10CT6C+11CT6C+12CT6C+13CT6C+14CT6C	9	1	
		6CT6C+7CT6C+8CT6C	6CT6C+7CT6C+8CT6C+9CT6C+10CT6C	12	1	
		15C+16C+17C+18C+19C+20C	15C+16C+17C+18C	13	1	
		7CT6C+8CT6C+9CT6C	14C+15C+16C+17C+18C	16	1	
		8CT6C+9CT6C	8CT6C+9CT6C+10CT6C	34	1	
	16189 Poly C	9C+10C+11C+12C+13C	9C+10C+11C+12C	15	1	
		4CT4C+5CT4C	3CT4C+5CT4C	17	1	
		9C+10C+11C+12C	10C+11C+12C	21	1	
		10C+11C	9C+10C+11C	31	1	
	514 CA	CA4+CA5	CA4	32	1	
	303 Poly C	7CT6C+8CT6C+9CT6C+10CT6C	5CT6C+6CT6C+7CT6C+8CT6C+9CT6C	8	1	
	16189 Poly C	9C+10C+11C+12C+13C	10C+11C+12C+13C			
	303 Poly C	8CT6C+9CT6C+10CT6C+11CT6C	7CT6C+8CT6C+9CT6C+10CT6C	26	1	
	16189 Poly C	5CT4C	4CT4C+5CT4C			
	303 Poly C	8CT6C+9CT6C+10CT6C+11CT6C+12CT6C	13C+14C+15C+16C+17C	27	1	
	16189 Poly C	9C+10C+11C+12C+13C	10C+11C+12C+13C			
	303 Poly C	6CT6C+7CT6C+8CT6C+9CT6C+10CT6C	8CT6C+9CT6C+10CT6C	33	1	
	514CA	CA4+CA5	CA4			
	**Subtotal**				**18**	**52.9**
**Total**					**18**	**52.9**

### Spectrum of Length Heteroplasmic Mutations

A gene scan (capillary electrophoresis) showed length heteroplasmies in 303 poly C, 16189 poly C and 514 CA repeats which were confirmed by TA cloning. The pattern of BT-specific length heteroplasmic mutation at 303 poly C region was distributed into 8CT6C+9CT6C+10CT6C+11CT6C, 7CT6C +8CT6C +9CT6C +10CT6C+11CT6C+12CT6C, 7CT6C+8CT6C, 8CT6C+9CT6C+10CT6C+11CT6C+12CT6C+13CT6C+14CT6C, 6CT6C+7CT6C+8CT6C+9CT6C+10CT6C, 15C +16C +17C +18C, 14C +15C +16C +17C +18C, 8CT6C+9CT6C+10CT6C (2.9%/each) (Table 1, Fig. 2). The pattern of BT-specific length heteroplasmic mutation at 514 (CA) repeats in HV2 regions disclosed 4(CA) repeats (2.9%) (Table 1). BT-specific length heteroplasmic mutation at 16189 poly C displayed four types of patterns: 9C +10C +11C +12C, 3CT4C+5CT4C, 10C +11C +12C, 9C +10C +11C (2.9%/each) (Table 1). Specimens that showed dissimilarities in length heteroplasmy between patient adjacent NT and BT were 23% (8 cases) for 303 poly C, 2.9% (1 case) for 514 (CA) repeats and 11% (4 cases) for 16189 poly C. Three patients had BT-specific length heteroplasmic mutations arising in both 303 poly C and 16189 poly C stretch regions. One patient had BT-specific length heteroplasmic mutations from both 303 poly C and 514 (CA) repeats (Table 1).

### mtDNA Copy Number

**Figure 3:**
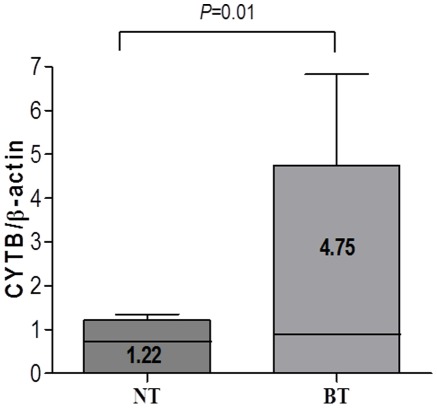
Significant increase in mtDNA copy number in Barrett’s metaplasia tissue. Quantitation of mtDNA copy number was calculated from the ratio of copy number (copies/µl) from mtDNA cytochrome b and nuclear ß-actin gene using real-time PCR. The average mtDNA copy number was significantly increased in Barrett’s metaplasia tissues (BT) compared to those from adjacent normal mucosal tissues (NT) (*P* = 0.01).

The mtDNA copy number (molecules/ul) from BT and adjacent NT showed 4.75±12.14, 1.22±0.78 (Fig. 3). The mtDNA copy number was significantly increased in BT than adjacent NT (*P* = 0.01).

### Hydrogen Peroxide Content

**Figure 4:**
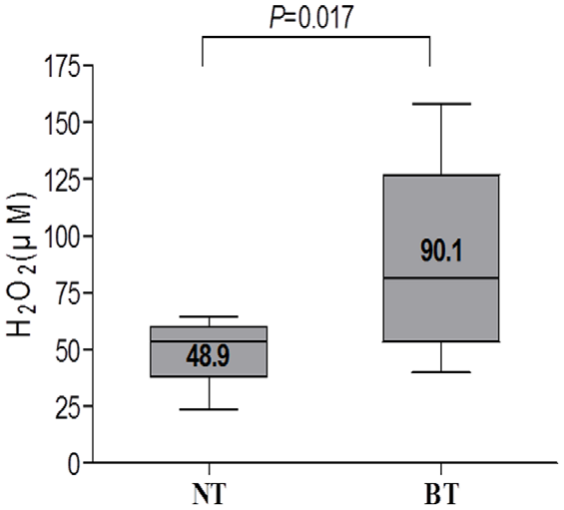
Production of high level of reactive oxygen species in Barrett’s metaplasia tissue. Quantitative determination of reactive oxygen species (hydrogen peroxide) from supernatants of Barrett’s metaplasia tissue (BT) and adjacent normal mucosal tissue (NT) was measured by commercial kit (Bioxytech H_2_O_2_-560). The level of hydrogen peroxide was significantly elevated in supernatants from BT compared with those from adjacent NT (*P* = 0.017).

Hydrogen peroxide was measured from BT and corresponding adjacent NT specimen to investigate the cause of BT-specific mtDNA mutations founded in the current study. The level of hydrogen peroxide significantly elevated in supernatants from BT (90.1±50.7 lM/mg protein) compared with those from adjacent NT (48.9±17.6 lM/mg protein) (Fig. 4). The level of hydrogen peroxide in the BT was significantly higher than those from the corresponding samples (*P* = 0.017).

### Quantitation of mtDNA Large (4977 bp) Deletion

**Figure 5:**
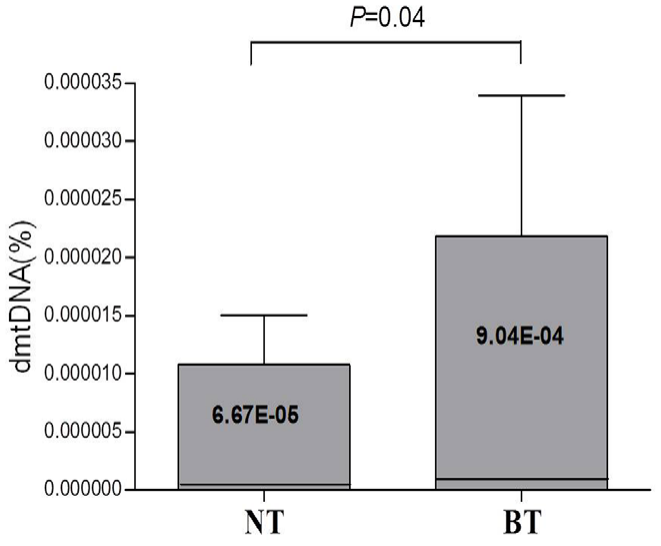
Increase of mtDNA large (4977 bp) deletions in Barrett’s metaplasia tissue. The amounts of mtDNA large deletions were significantly increased in Barrett’s metaplasia tissue (BT) compared with those from matched adjacent normal tissue (NT).

mtDNA large (4977 bp) deletion in BT and matched adjacent NT were quantified using gene scan and real-time PCR which were developed in the current study. The amounts of mtDNA large deletion were significantly increased in BT compared with those from adjacent NT (*P* = 0.04) (Fig. 5).

### Functional Alteration of Respiratory Chain Enzyme Complex Activity in Barrett’s Tissue

The activity of mitochondrial respiratory chain enzyme complexes in Barrett’s tissue was impaired in comparison with those of the adjacent normal mucosa: Decreased enzyme activity in respiratory chain complexes I, II and III was observed in Barrett’s tissue (Table S3).

## Discussion

Carcinogenesis is a long-term, multistep process driven by genetic and epigenetic changes in susceptible cells, which gain a selective growth advantage and undergo clonal expression [11]. BE is a premalignant condition that predisposes to the development of EA. It is detected on endoscopy and confirmed histologically by the presence in the lower esophagus of a metaplastic mucosa, the so-called specialized epithelium, which resembles incomplete intestinal metaplasia in the stomach. The similarities with incomplete intestinal metaplasia is present on histology, mucin histochemistry and immunohistochemistry with various differentiation markers (cytokeratins and MUC antigens). On morphology, the carcinogenetic process of Barrett’s mucosa progresses through increasing grades of epithelial dysplasia. Dysplasia, a synonym of intraepithelial neoplasia, is the only marker that can be used at the present time to delineate a population of patients at high risk of cancer.

Although high-grade dysplasia is considered a precursor to invasive adenocarcinoma, detection of this abnormal mucosa remains controversial and currently requires esophagoscopy with biopsy. It has been suggested that epithelial markers, such as increased activity of mucosal ornithine decarboxylase, sulfomucin production, nuclear DNA aneuploidy, and recently molecular analysis, have also been proposed to identify patients at increased risk for malignant degeneration [12].

Similar to other malignant tumors, carcinogenesis of EA is characterized by several genetic and epigenetic alterations with genetic instability (e.g., loss of heterozygote and/or ploidy), abnormal expression profiles of oncogenes (e.g., c-myc, VEGF and its receptors) and alterations in the DNA-methylation pattern (e.g., hypermethylation of p16, TIMP-3, DAPK, SOCS1 and SOCS3, and hypomethylation of CDX1. Molecular changes are early events in Barrett’s pathogenesis. The prevalence of these molecular alterations increases during the malignant transformation from BE to dysplasia and EA. Although for the majority of hitherto identified genes, the predictive or prognostic value remains unclear. Nearly all of the new markers are not yet validated in prospective controlled or randomized studies [13].

Among the numerous molecular events that have been shown to play a role in the neoplastic transformation of Barrett’s mucosa, only changes in DNA ploidy, increased proliferation, and alterations of the p53 gene have been suggested to be of potential help in the carcinogenesis [14]. As mentioned in introduction, mtDNA mutation has been regarded as oncogenic factors [15]. However, the study on mitochondrial dysfunction and mtDNA mutation was paucity in BE. The mitochondrion is an important microrganelle that produces the energy for cell development, differentiation and cell growth by inducing apoptosis. Human mtDNA contains 16,569 base pairs and represents 0.1∼1.0% of the total genomic DNA. A mammalian cell contains approximately 1,000 mitochondria, and each mitochondrion contains 2 to 10 DNA copies. There is a 10∼20 fold greater susceptibility of mtDNA to genetic mutation because mtDNA does not contain introns in comparison to nuclear DNA, the mtDNA repair system is inefficient and there is a higher exposure to ROS produced in the process of ATP synthesis. ROS plays an important role as potential carcinogens in the pathogenesis of GERD, BE and gastritis [16]. Oxidative damage has long been related to mucosal damages of gastrointestinal tracts and their ensuing carcinogenesis. In spite of treatment with anti-secretory medications for reflux esophagitis, considerable portions of patient did not achieve the complete mucosal healings or suffered from sustaining symptoms or development of dread complication like BE, suggesting other damaging factors or impaired mucosal resistance are also involved in their pathogenesis [17], [18].

BE occurs due to chronic inflammation which is mainly caused by GERD. An increase in ROS production frequently happens in chronic inflammatory cells and tissue. Thus, the current study attempted to detect BT-specific mtDNA alterations with the assumption that mtDNA aberrations are caused by ROS abundantly produced in chronically inflamed BT. mtDNA mutations in the current study were detected exclusively in BT samples but not in adjacent NT. These mtDNA mutations were observed as base substitutions and length heteroplasmy in the control region containing. The oxidative stress elicited by chronic inflammation increases the number of mtDNA mutations in BT and might correlate with a precancerous status [19]. The level of ROS was significantly higher in the supernatants obtained from BT compared with those from the adjacent NT. This high level of ROS might damage the mitochondria, leading to mtDNA mutations. mtDNA control region contains the mtDNA production regulating fraction and HV region known as a ‘hot spot’ of gene mutation in various degenerative diseases and tumors [20].

Recent reports have showed that severe morphological and functional alterations of cells and tissue are accompanied by a decrease of mtDNA copy number [6]. This is known to be a consequence of mtDNA mutation in the region near the replication origin of mtDNA. In contrast, it has been reported that the number of mtDNA copies increases if ATP synthesis is hindered by aging or chronic inflammation [6]. Concurrent with these reports, the number of mtDNA copies measured in BT was approximately four times higher than those in corresponding adjacent NT in the current study.

Recently one study reports that the frequency of specimens with the 4977 bp deletion increased in relation to the degree of dysplasia, so the mtDNA 4977 bp deletion may be useful as a biomarker to detect the severity [9]. The current study also confirms that the amounts of mtDNA large deletion were significantly increased in BT compared with those from either matched adjacent NT.

Importantly, mutations in the control region might alter the rate of DNA replication by modifying the binding affinity of significant trans-activating factors. These mtDNA alterations in BT might further impair a respiratory chain defect resulting in increasing the mtDNA copy number to compensate for the deficiency in ATP. During this perturbation, mitochondria might produce a large amount of ROS, which causes the vicious cycle observed in other chronic inflammatory diseases [17].

BT-specific mtDNA mutations frequently occurred in both the mtDNA control and minisatellite regions due to excessive production of ROS. High level of ROS in BT may contribute to development of mtDNA mutations, which may play a crucial role in pathophysiology of BE and furthermore progression to EA. Although many risk factors are considered in BE and EA, antioxidant treatment seems to be the therapeutics in the prevention or treatment of BE and moreover it plays the preventive role in disease progression.

## Materials and Methods

### Patient Specimens and Tissue Staining

**Figure 6:**
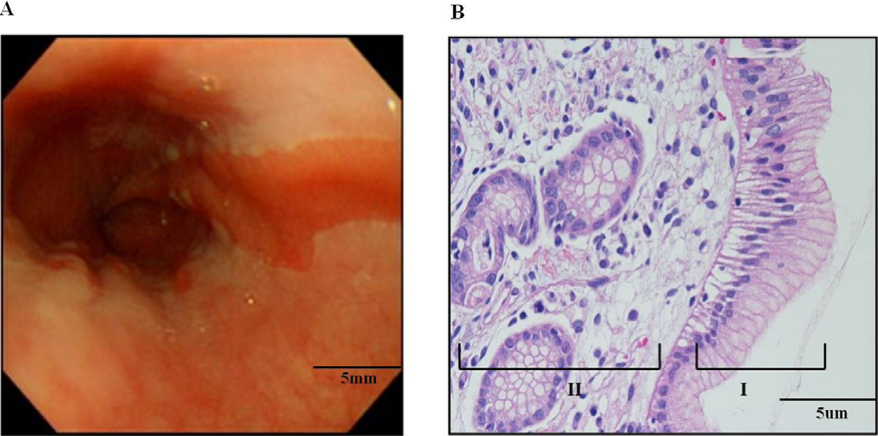
Endoscopic finding and histological examination of Barrett’s metaplasia. Endoscopic finding of esophagus shows irregular Z-line, elongated salmon patched tongue like projection of distal columnar epithelium (A). Histological examination (x200) of esophageal lesion reveals surface glandular epithelium with tall columnar cells punctuated by round to oval goblet cells (I). The columnar epithelium resembles intestinal epithelium but is not identical in nature. Barrett’s metaplasia is also characterized by universal presence of chronic inflammation (II) (B).

Thirty-four patients were enrolled after receiving Institutional Review Board approval and informed consent. Matched Barrett’s metaplasia tissue (BT) and adjacent normal mucosal tissue (NT) was obtained during the endoscopic procedure (Table S1). A diagnosis of BE was based on findings of endoscopy and specialized intestinal metaplasia on Hematoxylin and Eosin (H&E) stained tissue. Following standard tissue preparation, tissue was embedded in paraffin and was sliced with a microslicer. Sliced tissue was embedded into slides and stained with H&E (Fig. 6).

### Direct Sequencing of mtDNA Control Region

BT was obtained in a test tube containing 5 ml phosphate buffered saline (PBS, pH 7.4), sliced into small pieces with a sterilized mesh, and dissolved in protease K and tissue lysis buffer solution, from which DNA was extracted. Extracted total DNA was then dissolved in TE buffer (10 mM Tris-HCl, 1 mM Na_2_EDTA, pH 8.0) and was quantified with the use of a spectrophotometer. Published protocol was used to amplify and sequence mtDNA control region [20]. The mtDNA sequences obtained were analyzed using the Revised Cambridge Reference Sequence (http://www.mitomap.org/), Blast2 program (http://www.ncbi.nlm.nih.gov/blast/bl2seq/wblast2.cgi) and MitoAnalyzer (http://www.cstl.nist.gov/biotech/strbase/mitoanalyzer.html) to determine mtDNA aberrations (Table S2).

### Determination of mtDNA Length Heteroplasmies in the Control Region

mtDNA minisatellites are located in the mtDNA non-coding hypervariable regions (HV) 1 and 2. Size-based amplified product separation by capillary electrophoresis based on published protocol was performed in order to detect length heteroplasmic mutations of 16189 poly C (^16184^CCCCCTCCCC^16193^, 5CT4C), 303 poly C (^303^CCCCCCCTCCCCC^315^, 7CT5C) and 514 (CA) repeat (^514^CACACACACA^523^, (CA)5 repeats) [20], [21] (Table S2).

### TA Cloning for Confirmation of Length Heteroplasmic Mutations

The procedure including a set of primer pairs and PCR conditions was carried out according to the published protocol [22]. Each purified PCR product of 303 poly C, 16189 poly C and 514 (CA) repeat was inserted into pGEM-T Easy Vector (Promega, Madison, WI, USA). Competent *E. coli* (JM 109 cells) were then transformed with the plasmids containing the PCR product inserts. White colonies (8 to 12) including recombinant plasmids were selected and cultured. After plasmids extraction, length heteroplasmic mutations were determined by DNA sequencing (Table S2).

### Quantification PCR for Determination of mtDNA Copy Number

To generate a standard curve for quantification of mtDNA, purified PCR product of CYTB and β-actin gene as internal standard were inserted into pGEM-T easy vector and *E. coli* JM 109 cells (Promega) were transformed in order to obtain recombinant plasmids. A mixture of 25 µl containing 12.5 µl of 2×Quantitect SYBR green PCR master mix (Qiagen, Valencia, CA, USA), 400 µM CYTB primers F14909 (5′-TACTCACCAGACGCCTCAACCG-3′) and R15396 (5′-TTATCGGA ATGGGAGGT GATTC-3′) and 6 ng of total DNA was used for PCR with the Rotor-Gene real-time centrifugal DNA amplification system (Corbett Research, Sydney, Australia). For PCR, hot start reactions at 50°C for 2 min and at 95°C for 15 min were followed by 35 cycles of 20 sec at 94°C, 30 s at 56°C, 30 s at 72°C and a melting reaction with a decrease of 1°C per cycle between 72°C and 92°C. The mtDNA copy number was calculated using the following formula: {X µg/µl plasmid DNA/4419 (plasmid length)×660}×6.022×10^23^ =  Y molecules/ul. The X represents the concentration of plasmid DNA and the Y represents copy number. The mtDNA copy number was reported as the ratio of CYTB and β-actin gene.

### Quantitative Determination of Hydrogen Peroxide

BT and adjacent NT were homogenized in 500 µl of ice-cold deionized water. An aliquot of homogenate was taken for protein measurement. After centrifugation at 2000×g for 15 min, an aliquot of 200 µl from supernatant was transferred to new tube. Hydrogen peroxide content (lM/mg protein) from supernatant is measured using the commercial kit (Bioxytech_ H_2_O_2_-560TM, OXIS International, Foster City, CA, USA) according to the manufacturer’s instructions.

### mtDNA Large(4977 bp) Deletion

For generation of a fluorescent labeled 113 bp fragment of the undeleted mtDNA primers int1F-3448(5′-HEX-CCCTTCGCTGACGCCATA-3′) and int2R-3560 (5′-AGTAGAAGAGCGATGGTGAGAGC-3′) were used. Primers del1F-8395 (5′-HEX-CACCATAATTACCCCCATACTCCTTA-3′) and del2R-13494 (5′-GAGGAAAGG TATTCCTGCTAATGC-3′) were designed to flank the deletion breakpoints and were used to amplify a fluorescent labeled 123 bp amplicon of deleted mtDNA. For PCR, a 50 µl mixture containing 50 ng DNA, 200 µM dNTPs, 25 pmol primary primers, 2.5 U Taq DNA polymerase (TaKaRa LA Taq) and 5 µl 10× buffer. An initial denaturation step at 96°C for 5 min was followed by 20(int) cycles and 45(del) cycles of 15 s at 95°C, 20 sec at 60°C, 20 sec at 72°C and a 5 min extension step at 72°C. Specific fragments for intact and deleted mtDNA were amplified in two different PCR reactions in a thermal cycler (GeneAmp 2400, Applied Biosystems, Weiterstadt, Germany). After PCR was finished, a 1 µl aliquot of each PCR product and 0.5 µl of the internal size standard GS500 (Applied Biosystems) labeled with the fluorescent dye ROX (Applied Biosystems) were added to deionized formamide. Denaturation was performed at 96°C for 10 min followed by a cooling step at −22°C for 2 min. Denatured PCR products were separated by capillary electrophoresis using the ABI Prism 3130XL genetic analyzer (Applied Biosystems). When the run was completed, specific fragments were displayed as peaks in an electropherogram using the Gene Scan Analysis Software 3.1.

Quantitative PCR was conducted with a Rotor-Gene real-time centrifugal DNA amplification system (Corbett Research), at a final reaction volume of 25 µl containing 12.5 µl of 2×QuantiTect SYBR Green PCR master mix (Qiagen), DW, 10 pmol each of the forward primer del-lF (5′-CACCATAATTACCCCCATACTCCTTA-3′) and reverse primers del-2R (5′ GAGGAAAGGTATTCCTGCTAATGC-3′) and other mixture int-1F (5′-CCCTTCGCTGACGCCATA-3′), int-2R (5′-AGTAGAAGAGCGATGGTGAGAGC-3′) for 2 µl of template DNA. After denaturation at 95°C for 15 min, the reaction mixture was cycled 45 cycles at 95°C for 15 sec, 58°C for 30 sec and 72°C for 90 sec. PCR was performed for 45 cycles (deletion primer-1F/2R) and 20 cycles (intact primer-1F/2R). The percentage of the 4977 bp deletion was calculated according to the following formula: f (dmtDNA)  =  A_dmt_/A_mt_×1.9608^CDmt^/1.9613^CDdmt^×123/113. The followings are abbreviations and explanations for aforementioned formula: f (dmtDNA), frequency of the 4977 bp deletion; A_dmt_, value of peak area of deletion-specific peak; A_mt_, value of peak area of specific peak of intact mtDNA; CDdmt, number of cycles necessary to detect the 4977 bp deletion; CDmt, number of cycles necessary to detect intact mtDNA.

### Determination of Activity of Mitochondrial Respiratory Chain Enzyme Complexes

Cell homogenates were diluted to 1 g/L total protein with 20 mmol/L potassium phosphate buffer (pH 7.2) before respiratory chain complex analysis. Assay conditions were based on previously published spectrophotometric methods [23].

### Statistical Analysis

Descriptive data were expressed as mean ± standard deviation. The non-parametric Kruskal-Wallis test was used for the determination of statistical difference in the mtDNA copy number and hydrogen peroxide content between three specimens (BT and adjacent NT). *P*-value that is lower than 0.05 was considered as statistically significant.

## Acknowledgments

The authors would like to thank all patients who participated in the study.

## Supporting Information

Table S1 **Age and sex distribution of subjects.**
(DOC)

Table S2 **Primers for mtDNA control region PCR, direct sequencing, and gene scan.**
(DOC)

Table S3 **Respiratory chain complex I- IV activities in primary Barrett’s metaplasia tissues and adjacent normal tissues.**
(DOC)
