# Supplementary material for: Frequent Occurrence of Mitochondrial DNA Mutations in Barrett’s Metaplasia without the Presence of Dysplasia
Source: PLoS One. 2012 May 22;7(5):e37571. doi: 10.1371/journal.pone.0037571 (PMC3358277; doi:10.1371/journal.pone.0037571)
Supplement: Table S1 — Age and sex distribution of subjects. (DOC) [file pone.0037571.s001.doc]

**Table S1. Age and sex distribution of subjects**

| No. | Sex/Age | Sample (tissue) | | No. | Sex/Age | Sample (tissue) | |
| --- | --- | --- | --- | --- | --- | --- | --- |
|  |  | NT | BT |  |  | NT | BT |
| 1 | M/73 | O | O | 18 | M/50 | O | O |
| 2 | F/70 | O | O | 19 | F/37 | O | O |
| 3 | M/56 | O | O | 20 | M/59 | O | O |
| 4 | F/81 | O | O | 21 | M/63 | O | O |
| 5 | M/76 | O | O | 22 | M/80 | O | O |
| 6 | M/60 | O | O | 23 | F/67 | O | O |
| 7 | M/61 | O | O | 24 | F/68 | O | O |
| 8 | M/83 | O | O | 25 | M/53 | O | O |
| 9 | F/81 | O | O | 26 | F/67 | O | O |
| 10 | F/68 | O | O | 27 | M/66 | O | O |
| 11 | F/66 | O | O | 28 | M/54 | O | O |
| 12 | M/60 | O | O | 29 | M/67 | O | O |
| 13 | F/67 | O | O | 30 | M/59 | O | O |
| 14 | M/53 | O | O | 31 | M/50 | O | O |
| 15 | M/78 | O | O | 32 | M/67 | O | O |
| 16 | M/78 | O | O | 33 | M/66 | O | O |
| 17 | M/77 | O | O | 34 | M/68 | O | O |

NT, adjacent normal mucosal tissue; BT, Barrett’s metaplasia tissue.
