# Supplementary material for: Frequent Occurrence of Mitochondrial DNA Mutations in Barrett’s Metaplasia without the Presence of Dysplasia
Source: PLoS One. 2012 May 22;7(5):e37571. doi: 10.1371/journal.pone.0037571 (PMC3358277; doi:10.1371/journal.pone.0037571)
Supplement: Table S2 — Primers for mtDNA control region PCR, direct sequencing, and gene scan. (DOC) [file pone.0037571.s002.doc]

**Table S2. Primers for mtDNA control region PCR, direct sequencing, and gene scan**

| **mtDNA Segment** | **Primer sequence (5' to 3') for PCR** | | | **Sequencing primers (5' to 3')** | | |
| --- | --- | --- | --- | --- | --- | --- |
| **Control region**  **1.12 kb (np 16024-16569;**  **np 1-576)** | F15971 | TTAACTCCACCATTAGCACC | | F15971 | TTAACTCCACCATTAGCACC | |
|  |  |  |  | R48 | GCATGGAGAGCTCCCGTGAGTGG | |
|  | R611 | CAGTGTATTGCTTTGAGGAGG | | F15 | CACCCTATTAACCACTCACG | |
|  |  |  |  | R611 | CAGTGTATTGCTTTGAGGAGG | |
| **Minisatellite markers** | **Location**  **(bp)** | **Size (bp)** | **Primer sequences (5' to 3') for genescan** | | | **Change induced** |
| **303 poly C** | D-loop  (303-315) | 109-118 | F: CTTTCCACACAGACATCATAAC R: ATCTGGTTAGGCTGGTGTTAG | | | None |
| **16184 poly C** | D-loop (16184-16193) | 102-105 | F: CTTGACCACCTGTAGTACATA R: GGAGTTGCAGTTGATGTGTGA | | | None |
| **514(CA)**  **repeat** | D-loop  (514-523) | 82-86 | F: CCCATACTACTAATCTCATCAA R.: TTTGGTTGGTTCGGGGTATG | | | None |
| **3566 poly C** | ND1  (3566-3571) | 84 | F: CCGACCTTAGCTCTCACCAT R: AATAGGAGGCCTAGGTTGAG | | | Transcription stop |
| **12385 poly C** | ND5  (12385-12390 ) | 100 | F: CACCCTAACCCTGACTTCC R: GGTGGATGCGACAATGGATT | | | Transcription stop |
| **12418 poly A** | ND5  (12418-12425) | 100 | F: CACCCTAACCCTGACTTCC R: GGTGGATGCGACAATGGATT | | | Transcription stop |
