# Supplementary material for: Frequent Occurrence of Mitochondrial DNA Mutations in Barrett’s Metaplasia without the Presence of Dysplasia
Source: PLoS One. 2012 May 22;7(5):e37571. doi: 10.1371/journal.pone.0037571 (PMC3358277; doi:10.1371/journal.pone.0037571)
Supplement: Table S3 — Respiratory chain complex I- IV activities in primary Barrett’s metaplasia tissues and adjacent normal tissues. (DOC) [file pone.0037571.s003.doc]

**Table S3. Respiratory chain complex I- IV activities in primary Barrett’s metaplasia tissues and adjacent normal tissues**

| Samples | RCC (mean ± SD) activity, Vmax (mili-units per min) | | |
| --- | --- | --- | --- |
|  | I | II+III | IV |
| Barrett’s tissue | 4.533 ± 1.452 | -3.255 ± 0.534 | -0.037 ± 0.133 |
| Normal mucosa | 6.254 ± 1.258 | -4.932 ± 0.725 | 0.273 ± 0.321 |

RCC, respiratory chain enzyme complex.
